# LDAKM-EIoT: Lightweight Device Authentication and Key Management Mechanism for Edge-Based IoT Deployment

**DOI:** 10.3390/s19245539

**Published:** 2019-12-14

**Authors:** Mohammad Wazid, Ashok Kumar Das, Sachin Shetty, Joel J. P. C. Rodrigues, Youngho Park

**Affiliations:** 1 Department of Computer Science and Engineering, Graphic Era Deemed to be University, Dehradun 248 002, India; wazidkec2005@gmail.com; 2 Center for Security, Theory and Algorithmic Research, International Institute of Information Technology, Hyderabad 500 032, India; 3 Virginia Modeling, Analysis and Simulation Center, Center for Cybersecurity Education and Research, Department of Computational Modeling and Simulation Engineering, Old Dominion University, Suffolk, VA 23435, USA; sshetty@odu.edu; 4 Federal University of Piauí (UFPI), 64049-550 Teresina-Pi, Brazil; joeljr@ieee.org; 5 Instituto de Telecomunicações, 1049-001 Lisbon, Portugal; 6 School of Electronics Engineering, Kyungpook National University, Daegu 41566, Korea

**Keywords:** Internet of Things (IoT), edge computing, authentication, key management, security, AVISPA, NS2 simulation

## Abstract

In recent years, edge computing has emerged as a new concept in the computing paradigm that empowers several future technologies, such as 5G, vehicle-to-vehicle communications, and the Internet of Things (IoT), by providing cloud computing facilities, as well as services to the end users. However, open communication among the entities in an edge based IoT environment makes it vulnerable to various potential attacks that are executed by an adversary. Device authentication is one of the prominent techniques in security that permits an IoT device to authenticate mutually with a cloud server with the help of an edge node. If authentication is successful, they establish a session key between them for secure communication. To achieve this goal, a novel device authentication and key management mechanism for the edge based IoT environment, called the lightweight authentication and key management scheme for the edge based IoT environment (LDAKM-EIoT), was designed. The detailed security analysis and formal security verification conducted by the widely used “Automated Validation of Internet Security Protocols and Applications (AVISPA)” tool prove that the proposed LDAKM-EIoT is secure against several attack vectors that exist in the infrastructure of the edge based IoT environment. The elaborated comparative analysis of the proposed LDAKM-EIoT and different closely related schemes provides evidence that LDAKM-EIoT is more secure with less communication and computation costs. Finally, the network performance parameters are calculated and analyzed using the NS2 simulation to demonstrate the practical facets of the proposed LDAKM-EIoT.

## 1. Introduction

The Internet of Things (IoT) is a network of physical objects (for example, smart devices) such as smart vehicles, smart industrial monitoring machines, smart home appliances, and many more. Such objects are connected together to gather, process, rectify, and exchange relevant data over the Internet [1,2,3,4,5,6]. Furthermore, smart devices along with application programming interfaces (APIs) are used to connect and exchange data over the Internet. Each physical object (i.e., smart lighting system) has a provided IP address, which makes it capable to communicate (i.e., sending and receiving) over the network without human involvement. The shortage of IPv4 addresses resulted in designing the “IPv6 over Low-Power Wireless Personal Area Networks (6LoWPAN)”, which has significantly changed the IoT in order to increase the use of IPv6 by smart, as well as small scale objects [7]. The IoT communication environment is used in various types of applications, such as “smart health care”, “smart traffic monitoring”, and “smart homes”, to name a few. Edge computing introduced a new concept of computing. It has already become popular in industry, as well as academic research communities. It empowers many future technologies (for example, 5G, vehicle-to-vehicle and vehicle-to-cloud communications, augmented reality) by putting the connection in between end users and the cloud computing model and services [8,9,10]. Edge computing brings the utilities and services of cloud computing, which results in the faster processing and quicker response time of applications. Edge computing facilities the produced data of smart devices in the IoT environment to be processed nearby the location where it was generated in place of sending it across long routes to the “cloud” or “data centers” [10,11,12]. Though the edge based IoT environment provides many advantages over the traditional computing environment, at the same time, it has also the following security and privacy issues:

- The exchanged messages among different communicating parties (i.e., “IoT device”, “edge node”, and “cloud server”) should be protected against several known attacks (for example, “replay”, “man-in-the-middle”, “impersonation”, “offline or online password guessing”, and some other kinds of “bypassing attacks”).
- The edge node receives and processes the data, which are sent by the IoT devices. After the required processing, the edge node sends the data to the cloud server(s) for further processing and storage. Sometimes, the sent data are very critical and important, and any kind of disclosure of the data creates big trouble. Therefore, we need strong secure privacy preservation techniques to protect the data in the edge based IoT environment.
- As existing authentication protocols have security flaws that make them vulnerable to some known attacks (for example, “privileged insider attack”, “online and offline password guessing”, etc.), consequently, it becomes important to enhance the security of the authentication protocols, for instance in the case of “new device addition or revocation”, other communicating parties of the network should also be informed by the concerned (trusted) authority so that they can become aware about this activity and can update their memories accordingly [1,4]. Hence, it is an exigent task to provide a design for such a kind of protocol that supports “dynamic installation/update” without compromising the security of the system [4].
- In the edge based IoT environment, there is a possibility that some IoT devices may be “physically stolen/captured” by the adversary (A). After the physical capturing, A can use a “power analysis attack” [13] to obtain the data from the memory of the captured smart IoT devices. Later on, this drawn out information is used for other malicious works, such as deriving the “session key” among an IoT device and cloud server. A can replace the physical captured device with his/her own malicious device(s) that he/she has cloned in the laboratory. We should be more careful while going for the design of authentication and key management techniques in case some IoT devices are physically compromised so that there should not be any affect on the security of communication happening in the rest of the network [1,4].

As discussed earlier, the edge based IoT environment has several issues related to security and privacy. The existing authentication schemes have various “security flaws” that make them vulnerable to different known and unknown attacks. Some of them are not efficient from the computation and communication cost point of few. Hence, there is an essential requirement for a new “lightweight authentication and key management” scheme for the edge based IoT environment. Consequently, we design a new “lightweight authentication and key management” scheme for such an environment.

### 1.1. Contributions of LDAKM-EIoT

The contributions of this paper are many-fold in the following contexts:

- We propose a new “lightweight authentication and key management” scheme for edge based IoT environment (LDAKM-EIoT). In LDAKM-EIoT, we use various efficient operations such as bitwise “exclusive-OR (XOR)” and “one way collision resistant cryptographic hash functions”.
- LDAKM-EIoT is secure against different kinds of attacks by the help of “formal security verification” using the widely used AVISPA tool and also through other mathematical security analysis.
- The detailed comparative investigation among related existing schemes and LDAKM-EIoT evidences that LDAKM-EIoT achieves more security and additional functionality features and LDAKM-EIoT has also less communication and computation costs as compared to the other related schemes.
- The practical simulation study of LDAKM-EIoT is also executed with the help of the broadly used NS2 tool.

### 1.2. Paper Structure

The information about the “network model” and “threat model” of LDAKM-EIoT is discussed in Section 2. The brief discussion on the related existing authentication techniques is provided in Section 3. Different phases of LDAKM-EIoT are explained in Section 4. The security analysis of LDAKM-EIoT is conducted in Section 5. The formal security verification by the help of the AVISPA tool is done and explained in Section 6. The performance comparison among LDAKM-EIoT and other related existing authentication techniques is explained in Section 7. The impact of LDAKM-EIoT and other related schemes on network performance parameters is measured, analyzed, and then compared in Section 8 using the NS2 simulation. At last, the paper is concluded in Section 9.

## 2. Related Work

Wolf and Serpanos [14] explained different security issues of cyber-physical systems (CPS) and IoT systems. They discussed a security (safety) threat model for CPS and IoT systems. Ni et al. [15] explained the role of fog nodes in various IoT applications. After that, they examined several promising IoT applications as per these different roles.

Yeh et al. [16] demonstrated an “elliptic curve cryptography (ECC)” based user authentication mechanism for wireless sensor networks (WSNs). Their scheme did not achieve the mutual authentication property. To fix the problem of their scheme, Shi and Gong [17] proposed another improved “ECC based user authentication” scheme in WSNs. After that, Turkanovic et al. [18] came up with another user “authentication and key establishment” protocol for heterogeneous WSNs. Later on, their scheme was discovered to be insecure against “offline password guessing”, “offline identity guessing”, “smart card stolen”, “sensor node impersonation”, and “user impersonation” attacks. Moreover, their scheme did not support one of the essential properties, named as “mutual authentication” [19].

Khalil et al. [20] shed some light on the integration of WSNs in IoT. In the IoT environment, smart devices have limited computing and storage resources, like WSNs. It is also emphasized that some of the existing authentication techniques had serious security flaws as they were vulnerable to “impersonation”, “sensing node physical capture”, “replay”, “man-in-the-middle”, and “privileged insider” attacks [3,21].

Farash et al. [21] demonstrated a technique for “user authentication and key establishment” for heterogeneous WSNs, which can be applicable for IoT communication. Later on, Amin et al. [22] did cryptanalysis on the scheme of Farash et al. [21] and discovered that it was not secure against possible attacks such as “offline password guessing” by using the lost/stolen smart card, “session-specific temporary information leakage”, and “user impersonation” attacks. Furthermore, Amin et al. [22] proposed an improved version of their scheme to mitigate the security flaws of Farash et al.’s scheme [21]. Srinivas et al. [23] demonstrated that the scheme of Amin et al. [22] was insecure against “user impersonation”, “leakage of different keys”, “stolen smart card”, and “server spoofing” attacks. Srinivas et al. further presented an improved and enhanced version of their scheme for “user authentication” in multi-gateway WSNs applicable to the IoT environment. Jiang et al. [24] also discovered the security flaws in the scheme of Amin et al. [22] as it was not able to mitigate some attacks. To overcome these vulnerabilities in the scheme of Amin et al., Jiang et al. presented an improved scheme.

Hsieh and Leu [25] proposed a new technique to resolve the security weaknesses of the other schemes [26,27,28]. Later on, Wu et al. [29] performed crypto-analysis of Hsieh–Leu’s scheme and identified the vulnerabilities of their scheme as it was not secure against different attacks such as “offline guessing”, “user forgery”, “insider attack”, and “sensor node physical capturing”. In addition, their scheme was not secure against the session key security, and also, it did not provide the mutual authentication property. To resolve these problems, Wu et al. presented a new “user authentication” protocol for WSN, which was also applicable to IoT communication.

Li et al. [30] presented a device-to-device authentication protocol in the IoT environment. Their approach relied on public key techniques that needed public key encryption and decryption by the resource limited IoT smart devices. Though their approach maintained security, it demanded high communication and computation overheads from the IoT devices’ point of view.

Santos et al. [31] designed a “federated identity management (FIdM)” system in order to assist in improving privacy and user authentication in the IoT deployment, where an IoT device accesses services from a service provider. Their approach was lightweight as it required low computational cost due to symmetric cryptographic operations. They applied their proposal to a cashless toll system environment.

Gope and Sikdar [32] designed a “lightweight and privacy preserving two factor authentication” scheme in which physical security was included. In their proposal, an IoT device and a server mutually authenticated each other for accessing services in the IoT environment. However, their scheme needed more computational cost due to fuzzy extractor generation and reproduction functions [33].

In order to design an access control and security policy, Han and Kim [34] designed a mutual authentication scheme between IoT devices. Though their scheme was lightweight due to symmetric cryptographic operations, it did not preserve device anonymity and untraceability properties.

Group key management (GKM) is considered as another important security aspect in the IoT environment, which helps with assigning IoT devices into predefined groups in the network. After that, key management is essential within each group and also among various groups in order to improve efficiency and security in the IoT environment. For this issue, Kung and Hsiao [35] designed an efficient GKM policy in an IoT deployment. Their approach also permitted joining and leaving the devices in a group dynamically in the IoT environment. Their approach was lightweight as it relied on the “cryptographic hash function” and symmetric cryptographic operations.

Raza and Magnusson [36] pointed out that “unanimous consensus” is extremely essential in the IoT environment for cyber security purposes. They presented a lightweight adaptation of the “Internet Key Exchange Version 2 (IKEv2)” for the IoT deployment, called TinyIKE. With the help of TinyIKE, they were able to solve the key management issue for several IoT protocols by applying a single IKEv2 based approach. TinyIKE relies on certificates, elliptic curve cryptographic techniques, as well as symmetric cryptographic techniques.

Challa et al. [3] recommended an ECC based user authentication protocol for IoT applications that can be used in the coming future. However, Jia et al. [37] highlighted that Challa et al.’s method did not protect against impersonation attack and also it did not preserve the untraceability property. Moreover, Challa et al.’s scheme [3] was expensive in computation and communication. Recently, Malani et al. [38] presented a “certificate based device access control” scheme applied to IoT communication to mitigate the security problems and limitations of the existing authentication and access control protocols. Their scheme the preserved anonymity property.

Sharma and Kalra [39] proposed a lightweight user authentication protocol that can be applied to healthcare services in the cloud-IoT environment. However, their scheme was insecure against privileged-insider attack during the registration of medical professional, where the password of the medical professional was easily guessed by an attacker with the help of stolen smart card attack and registered information supplied by the medical professional to the gateway node. Moreover, their scheme did not provide the sensor node anonymity property and session key security under the Canetti and Krawczyk (CK) adversary model [40] (discussed in the threat model in Section 3.2). Zhou et al. [41] designed an authentication protocol that utilized IoT based architectures combined with cloud servers. Though their scheme was lightweight in nature, Martinez-Pelaez et al. [42] pointed out that Zhou et al.’s scheme [41] was vulnerable to various attacks, such as privileged-insider attack, man-in-the-middle attack, replay attack, and user impersonation attack. Furthermore, Martinez-Pelaez et al. also showed that Zhou et al.’s scheme failed to provide mutual authentication and secret key protection. To mitigate the security limitations mentioned in Zhou et al.’s scheme, Martinez-Pelaez et al. proposed an efficient authentication scheme.

As discussed above, most of the available methods for “authentication and key agreement” for communication in IoT and WSNs are insecure against different types of attacks. In addition, some of these techniques use heavy weight operations as they require more communication and computing resources. As a result, these existing techniques demonstrated for the “IoT environment” may not be much suitable for the authentication procedure in the edge based IoT environment. Consequently, we perceive that there is a requirement for a secure “authentication and key management” mechanism for the edge based IoT environment that should be lightweight. To fulfill this goal, we propose a new “lightweight authentication and key management” scheme for the edge based IoT environment that utilizes only an efficient “one way cryptographic hash” function and “bitwise XOR” operation.

## 3. System Models

The following two models are utilized to describe the working and usability of LDAKM-EIoT.

### 3.1. Network Model

**Figure 1:**
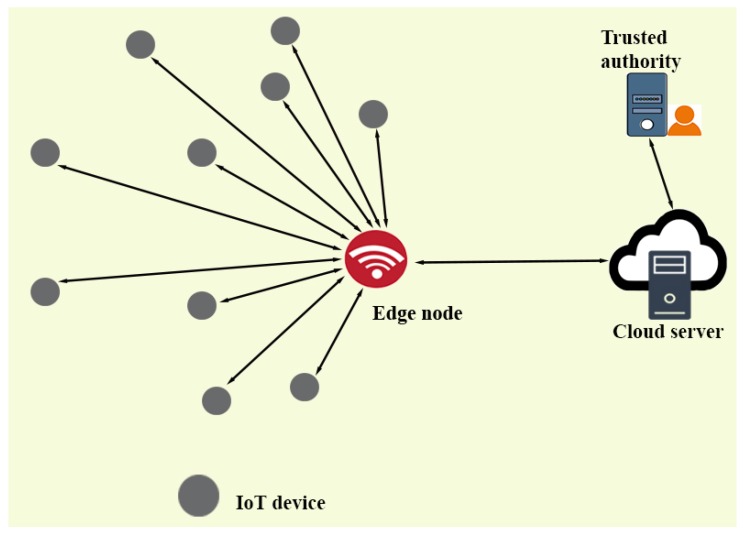
Network model of the edge based IoT environment.

The network model of LDAKM-EIoT is presented in Figure 1. In this figure, we have IoT devices, an edge node, which is a gateway to the Internet, the cloud server(s), and a trusted authority (TA). IoT devices can be deployed as per their applications (for example, smart health care, smart manufacturing, smart cities, and smart homes). The task of each IoT device is to collect and process information about some activity (i.e., level of fumes in a industrial plant) and then to send the data to the cloud server(s) through the edge node. At the cloud server, this will be stored and utilized for other processing and decision making tasks. In this model, IoT devices are resource constrained, whereas the edge node and cloud server are resource rich as they are able to do intensive computations and have more storage capacity and battery backup. The TA is responsible for the registration of different devices (i.e., IoT devices, edge nodes, and cloud servers). In such a kind of communication environment, we have to secure the communication between the IoT device and edge node and the edge node and cloud server. Since most of IoT devices are resource constrained, we preferred to use lightweight cryptographic operations (i.e., hash and XOR operations) in different exchanged messages’ computation. Consequently, to secure such kinds of communication, we urge a new “authentication and key management” protocol with lightweight operations. Using this scheme, the communicating entities can securely access the resources of other entities and also communicate securely.

### 3.2. Threat Model

The widely accepted “Dolev–Yao (DY) threat model” [43] was followed in the designing of LDAKM-EIoT. As per the guidelines of the DY model, two communicating entities communicate over an open (insecure) channel. The end-point entities such as “IoT devices” are not fully trusted in general. The existing network adversary (A) can eavesdrop, update, or delete the communicated messages as the channel is insecure. We also followed Canetti and Krawczyk’s adversary model, also named as the “CK adversary model” [40], which is the current de facto standard model in the designing and modeling of an “authenticated key agreement” security scheme. In the CK adversary model, A can have all the capabilities like the DY model. In addition to that, A can also compromise the secret credentials along with the “session states and session keys” during a session. Moreover, A can physical capture some IoT devices and extract the stored information from the IoT devices by the application of a sophisticated power analysis attack [13]. The obtained credentials can be further used to perform other kinds of malicious activities, such as the computation of “secret credentials” and the “session key”, and launch some attacks, such as “IoT device impersonation”, “replay”, “privileged-insider”, and “man-in-the-middle”. As per the information available in [44], we also assumed that the edge node (EN) was fully trusted and could not be compromised by A. Otherwise, if the EN was compromised, the entire network would be compromised. For such a purpose, we followed the method discussed by Bertino et al.’s protocol [45]. We assumed that the EN was equipped with a tamper resistant hardware device so that all the sensitive confidential information (for example, stored cryptographic keys) should be protected from A. Therefore, the application of a tamper resistant EN attained strong enough security in LDAKM-EIoT. Though it is also true that the attacks are possible on tamper resistant devices, A needs special equipment (device) to perform such an attack to acquire the stored information. This is because it is less expensive to install the EN than to use special equipment because A does not have any economic benefits to perform such an attack on a security scheme [45]. Moreover, we could secure the EN by putting it inside a physical locking system so that the physical capture of the EN will be difficult for A as compared to that for the unattended IoT smart devices. In addition, the “trusted authority (TA)” was also assumed to be a “fully trusted” entity of the network and that it would not be compromised, although the “cloud servers” were considered to be “semi-trusted” entities.

## 4. The Proposed Scheme: LDAKM-EIoT

In this section, we talk about the precise workings of the proposed scheme, called the “lightweight authenticated key management mechanism for the edge-based IoT environment (LDAKM-EIoT)”. The distinct phases of LDAKM-EIoT are provided in the upcoming part of the paper. It was also assumed that the different network entities (i.e., IoT device, edge node, cloud server) were synchronized with their clocks. It is mandatory to have this assumption while we go for the designing of an authentication mechanism for different networks [1,2,3,46,47,48]. We needed different notations for the designing of LDAKM-EIoT, which we summarize in Table 1 along with their significance.

### 4.1. Pre-Deployment Phase

This phase permitted the trusted authority (TA), a fully trusted entity in the network, to perform the registration of IoT devices, edge nodes, and cloud servers before they were installed in the deployment area. We assumed that $n_{d}$ number of IoT devices $D_{i}$ were associated with a particular edge node EN, and the real-time information could be accessed from the IoT devices to $n_{c}$ number of cloud servers $CS_{j}$, provided that a mutual authentication and key establishment happened successfully among the IoT devices and cloud servers with the presence of the edge node EN.

#### 4.1.1. Registration of IoT Devices

The TA firsts picked a distinct unique identity $ID_{D_{i}}$ for every IoT device $D_{i}$ and computed its corresponding pseudo identity as $RID_{D_{i}}$$=h(n||ID_{D_{i}})$, temporal credentials $TC_{D_{i}}=$$h(RID_{D_{i}}$$||ID_{EN}$$||ID_{TA}$||n$||RTS_{D_{i}})$, $AD_{i}$$=h(K_{EN-D_{i}}||$$ID_{D_{i}}$$||ID_{EN}\left|\right|$$RTS_{D_{i}})$ where $RTS_{D_{i}}$ is the registration timestamp for $D_{i}$, $K_{EN-D_{i}}$ is a 1024 bit shared secret key between each IoT device $D_{i}$ and edge nodes generated by the TA, where $i=1,2,\cdots n_{d}$, and $n_{d}$ is the total count of IoT devices. Moreover, $ID_{EN}$ is the identity of an edge node EN where the IoT devices need to authenticate a cloud server $CS_{j}$ with an IoT device $D_{i}$. $ID_{TA}$ is the identity of TA, and *n* is a 160 bit secret number of TA that is only disclosed to the TA. After that, TA generates a temporary identity $TID_{D_{i}}$ for each $D_{i}$. Note that temporal credentials are different for each IoT device $D_{i}$, which protects against various attacks including impersonation attack, in case any IoT device is physically compromised by an attacker. Lastly, the trusted authority stores the credentials {$RID_{D_{i}},$$TID_{D_{i}},$$TC_{D_{i}},$$AD_{i},$h(·)} in the memory of $D_{i}$ prior to its placement in the IoT network.

#### 4.1.2. Registration of Cloud Servers

The TA picks a distinct unique identity $ID_{CS_{j}}$ for each cloud server $CS_{j}$ and proceeds to calculate its pseudo identity as $RID_{CS_{j}}$=h(n$||ID_{CS_{j}})$ and $ACS_{j}$$=h(K_{EN-CS_{j}}$$||ID_{CS_{j}}$$||ID_{EN}$$||RTS_{CS_{j}})$ where $RTS_{CS_{j}}$ is the registration timestamp for $CS_{j}$ and j=1,2,$\cdots n_{c}$, $n_{c}$ are the number of cloud servers placed initially in the IoT network, while $K_{EN-CS_{j}}$ is a 1024 bit shared secret key of the edge node and cloud server $CS_{j}$ generated by the TA. Next, the TA stores the credentials {$RID_{CS_{j}},$$ACS_{j},$$ID_{CS_{j}},$h(·)} in the database of $CS_{j}$ before its placement in the network.

#### 4.1.3. Registration of Edge Node

The TA picks a distinct unique identity $ID_{EN}$ for edge node EN and stores previously computed information, as well as this information, that is $\langle \{(TID_{D_{i}},$$RID_{D_{i}},$$ID_{D_{i}},$$TC_{D_{i}},$$AD_{i})$$|1\leq i\leq n_{d}\},$$\{(ID_{CS_{j}},$$RID_{CS_{j}},$$ACS_{j})|1\leq j\leq n_{c}\},$h(·)〉, in the database of EN before its placement in the network.

**Figure 2:**
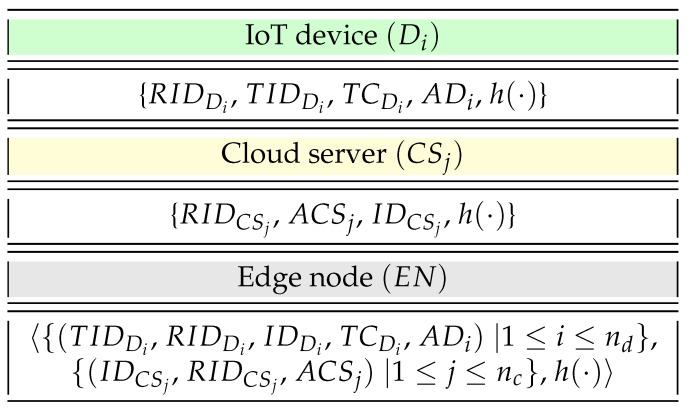
Credentials stored in $D_{i}$, $CS_{j}$, and EN during registration processes.

The registration process is summarized in Figure 2.

### 4.2. Authentication and Key Agreement Phase

The key management procedure helps to secure the authentication and key management among IoT devices and cloud servers by the involvement of a trusted edge node. The upcoming steps are essential to achieve this goal.

**A1.**  When an IoT device, say $D_{i}$, wants to initiate secure data transmission to a cloud server, first of all, $D_{i}$ needs to compute the following parameters. $D_{i}$ picks a random nonce $r_{d_{i}}$ and current timestamp $T_{1}$ and calculates $M_{1}$$=h(AD_{i}||T_{1})$$⊕r_{d_{i}}$ and $M_{2}$$=h(AD_{i}||$$T_{1}\left|\right|$$TID_{D_{i}}||RID_{D_{i}}\left|\right|$$TC_{D_{i}}$$\left|\right|r_{d_{i}})$. Next, $D_{i}$ sends the message $Msg_{1}=$$\{TID_{D_{i}},$$M_{1},$$M_{2},$$T_{1}\}$ to the edge node EN via an open channel.
**A2.**  After the arrival of message $Msg_{1}$ at time $T_{1}^{*}$, the EN checks the timeliness of $T_{1}$ with the verifying condition $|T_{1}-T_{1}^{*}|$≤ΔT. If it is valid, the EN fetches $RID_{D_{i}}$, $TC_{D_{i}}$, $AD_{i}$, and $ID_{D_{i}}$ corresponding to received $TID_{D_{i}}$ and computes $r_{d_{i}}$$=M_{1}⊕$$h(AD_{i}||T_{1})$, $M_{2}^{\prime }$$=h(AD_{i}$$\left|\right|T_{1}\left|\right|$$TID_{D_{i}}\left|\right|$$RID_{D_{i}}\left|\right|$$TC_{D_{i}}\left|\right|$$r_{d_{i}})$ and checks whether $M_{2}^{\prime }=M_{2}$. If it is valid, $D_{i}$ is authenticated by the EN and can access its resources to get access to a cloud server $CS_{j}$ selected by the EN. Next, the EN chooses a random nonce $r_{en_{1}}$ and current timestamp $T_{2}$, picks $ACS_{j}$ from its database corresponding to $ID_{CS_{j}}$, and computes $TID_{D_{i}}^{\prime }$$=TID_{D_{i}}⊕$$h(ACS_{j}||T_{2})$, $M_{3}$$=h(ACS_{j}||RID_{CS_{j}})$$⊕h(r_{en_{1}}$$\left|\right|T_{1}$$||AD_{i}$$||ID_{D_{i}}$$\left|\right|r_{d_{i}}$$||TC_{D_{i}})$ and $M_{4}$$=h(TID_{D_{i}}$$\left|\right|T_{2}$$||RID_{CS_{j}}$$||ACS_{j}$$||h(r_{en_{1}}$$\left|\right|T_{1}$$||AD_{i}$$||ID_{D_{i}}$$\left|\right|r_{d_{i}}$$||TC_{D_{i}}))$. After that, the EN sends the message $Msg_{2}=$$\{TID_{D_{i}}^{\prime },$$M_{3},$$M_{4},$$T_{2}\}$ to $CS_{j}$ via an open channel.
**A3.**  After the arrival of message $Msg_{2}$ at time $T_{2}^{*}$, $CS_{j}$ verifies the timeliness of $T_{2}$ by checking if $|T_{2}-T_{2}^{*}|$≤ΔT is valid. After successful verification of $T_{2}$, $CS_{j}$ computes $TID_{D_{i}}$$=TID_{D_{i}}^{\prime }⊕$$h(ACS_{j}$$\left|\right|T_{2})$, $h(r_{en_{1}}$$\left|\right|T_{1}$$||AD_{i}$$||ID_{D_{i}}$$\left|\right|r_{d_{i}}$$||TC_{D_{i}})=$$M_{3}⊕$$h(ACS_{j}||$$RID_{CS_{j}})$ and $M_{4}^{\prime }$$=h(TID_{D_{i}}$$\left|\right|T_{2}$$||RID_{CS_{j}}$$||ACS_{j}$$||h(r_{en_{1}}$$\left|\right|T_{1}$$||AD_{i}$$||ID_{D_{i}}$$\left|\right|r_{d_{i}}$$||TC_{D_{i}}))$. After that, $CS_{j}$ checks whether $M_{4}^{\prime }=M_{4}$ is satisfied. If it is valid, the EN is authenticated by $CS_{j}$. Otherwise, $CS_{j}$ halts the session with the EN immediately. Furthermore, $CS_{j}$ picks up a random nonce $r_{cs_{j}}$ and current timestamp $T_{3}$ and computes $M_{5}$$=r_{cs_{j}}⊕$$h(T_{2}||$$ACS_{j}\left|\right|$$RID_{CS_{j}})$. Then, $CS_{j}$ computes the session key shared with $D_{i}$ as $SK_{CS_{j}-D_{i}}$$=h(TID_{D_{i}}||$$T_{3}\left|\right|$$h(r_{en_{1}}$$\left|\right|T_{1}$$||AD_{i}$$||ID_{D_{i}}$$\left|\right|r_{d_{i}}$$||TC_{D_{i}})$$||h(r_{CS_{j}}$$||ACS_{j}$$||ID_{CS_{j}}$$\left|\right|T_{3}))$, $M_{6}$$=h(SK_{CS_{j}-D_{i}}$$\left|\right|T_{3})$, and $M_{7}$$=h(r_{cs_{j}}$$\left|\right|T_{2}$$\left|\right|T_{3}$$||ACS_{j}$$||RID_{CS_{j}})$. After computing these values, $CS_{j}$ sends the message $Msg_{3}=$$\{M_{5},$$M_{6},$$M_{7},$$T_{3}\}$ to the EN via an open channel.
**A4.**  After arrival of message $Msg_{3}$ at time $T_{3}^{*}$, the EN verifies the timeliness of $T_{3}$ by checking if $|T_{3}-T_{3}^{*}|$≤ΔT is valid. Upon successful validation of $T_{3}$, the EN calculates $r_{cs_{j}}$$=M_{5}⊕$$h(T_{2}$$||ACS_{j}$$||RID_{CS_{j}})$ and $M_{7}^{\prime }$$=h(r_{cs_{j}}||$$T_{2}\left|\right|$$T_{3}\left|\right|$$ACS_{j}\left|\right|$$RID_{CS_{j}})$. Next, the EN checks whether $M_{7}^{\prime }=M_{7}$, and if it holds, $CS_{j}$ is authenticated by the EN. Otherwise, the EN halts the session with $CS_{j}$ instantly. Furthermore, the EN selects a random nonce $r_{en_{2}}$ along with current timestamp $T_{4}$ and computes $M_{8}=$$h(AD_{i}||TC_{D_{i}})$$⊕h(r_{en_{2}}$$\left|\right|T_{4})$, α$=h(RID_{D_{i}}$$||TC_{D_{i}}$$\left|\right|T_{4})$$⊕h(r_{en_{1}}$$\left|\right|T_{1}$$||AD_{i}$$||ID_{D_{i}}$$\left|\right|r_{d_{i}}$$||TC_{D_{i}})$, β=$h(r_{CS_{j}}||$$ACS_{j}\left|\right|$$ID_{CS_{j}}$$\left|\right|T_{3})$$⊕h(h(r_{en_{2}}$$\left|\right|T_{4}))$$||AD_{i}||TC_{D_{i}})$ and $M_{9}=$$h(M_{6}||$$M_{8}\left|\right|$$h(r_{en_{2}}$$\left|\right|T_{4})$$\left|\right|T_{3}$$\left|\right|T_{4})$. After computing these values, the EN picks a new temporary identity $TID_{D_{i}}^{new}$ for $D_{i}$ and calculates $M_{10}$$=h(M_{9}$$\left|\right|T_{3}$$\left|\right|T_{4}$$||AD_{i}$$||TC_{D_{i}}$$||TID_{D_{i}})$$⊕TID_{D_{i}}^{new}$. The EN also replaces $TID_{D_{i}}$ with $TID_{D_{i}}^{new}$ in its database. Finally, the EN sends the message $Msg_{4}=$$\{M_{8},$α,β,$M_{9},$$M_{10},$$T_{3},$$T_{4}\}$ to EN via an open channel.
**A5.**  After arrival of message $Msg_{4}$ at time $T_{4}^{*}$, $D_{i}$ verifies the timeliness of $T_{4}$ by checking if $|T_{4}-T_{4}^{*}|$≤ΔT is valid. After successful validation of $T_{4}$, $D_{i}$ calculates $h(r_{en_{2}}$$\left|\right|T_{4})=$$M_{8}⊕$$h(AD_{i}||TC_{D_{i}})$, $h(r_{en_{1}}$$\left|\right|T_{1}$$||AD_{i}$$||ID_{D_{i}}$$\left|\right|r_{d_{i}}$$||TC_{D_{i}})=$α⊕$h(RID_{D_{i}}$$||TC_{D_{i}}$$\left|\right|T_{4})$, $h(r_{CS_{j}}||$$ACS_{j}\left|\right|$$ID_{CS_{j}}$$\left|\right|T_{3})=$β⊕$h(h(r_{en_{2}}$$\left|\right|T_{4}))$$||AD_{i}||TC_{D_{i}})$, the shared session key with $CS_{j}$ as $SK_{D_{i}-CS_{j}}$$=h(TID_{D_{i}}||$$T_{3}\left|\right|$$h(r_{en_{1}}$$\left|\right|T_{1}$$||AD_{i}$$||ID_{D_{i}}$$\left|\right|r_{d_{i}}$$||TC_{D_{i}})$$||h(r_{CS_{j}}$$||ACS_{j}$$||ID_{CS_{j}}$$\left|\right|T_{3}))$. In addition, $D_{i}$ also calculates $M_{6}^{\prime }$$=h(SK_{D_{i}-CS_{j}}$$\left|\right|T_{3})$ and $M_{9}^{\prime }=$$h(M_{6}^{\prime }||$$M_{8}\left|\right|$$h(r_{en_{2}}$$\left|\right|T_{4})$$\left|\right|T_{3}$$\left|\right|T_{4})$. Then, $D_{i}$ checks whether $M_{9}^{\prime }=M_{9}$, and if it is legitimate, the EN, as well as $CS_{j}$ are authenticated by $D_{i}$, and the computed session key is treated as the valid one. Thus, both $D_{i}$ and $CS_{j}$ will maintain the same computed shared session key $SK_{D_{i}-CS_{j}}$$\left(=SK_{CS_{j}-D_{i}}\right)$ for secret communications. Moreover, $D_{i}$ calculates the new temporary identity as $TID_{D_{i}}^{new}$$=M_{10}⊕$$h(M_{9}$$\left|\right|T_{3}$$\left|\right|T_{4}$$||AD_{i}$$||TC_{D_{i}}$$||TID_{D_{i}})$ and will use this new identity in all its future communications with the EN and $CS_{j}$. It is worth noticing that the new temporary identity generation mechanism makes the proposed scheme prevent the traceability attack.

**Figure 3:**
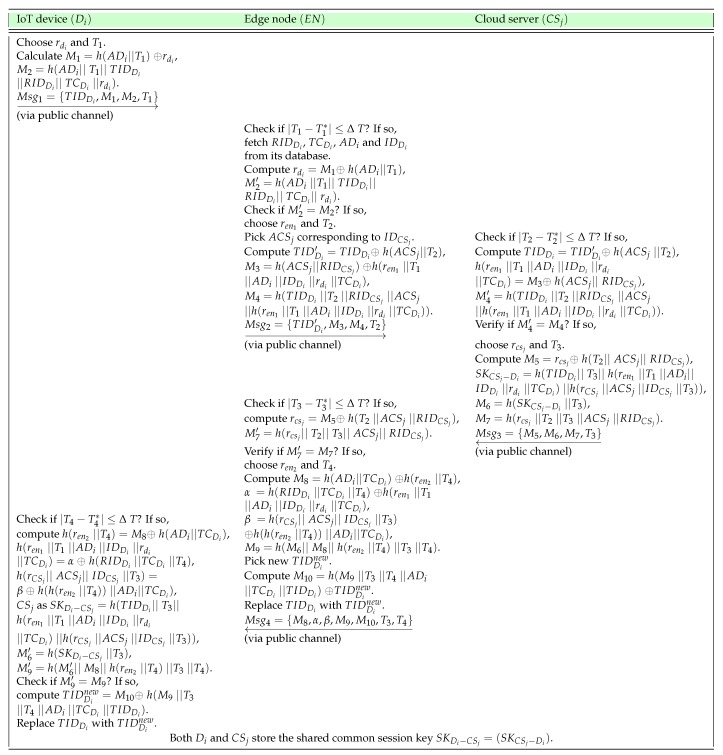
Abridging of the authentication and key agreement phase.

Finally, the above phase is abridged in Figure 3.

**Remark** **1** (Protection for synchronization attack). *We may consider a situation where the message ${\mathrm{Msg}}_{4}$ has been tampered with or there is a communication error that occurs so that an IoT device cannot receive the parameter $M_{10}$, as well as the new temporary identity $TID_{D_{i}}^{new}$. In order to resolve this problem, we contemplated a possible solution suggested in [49], where an IoT device needs to maintain a set of l shadow identities SID= {${\mathrm{sid}}_{1},$${\mathrm{sid}}_{2},$⋯,${\mathrm{sid}}_{l}$}. In this context, when an IoT device, say $D_{i}$, could not receive the message $Msg_{4}$ within a specific time period (maximum round-trip time), it needs to select one of the unused shadow identities, say ${\mathrm{sid}}_{j}\in \mathrm{SID}$, and then send it within the message $Msg_{1}$. When the EN receives the ${\mathrm{sid}}_{j}$, it can generate a new TIDand send it securely to the device $D_{i}$. By using this method, we can address the synchronization attacks without compromising the privacy of an IoT device in the proposed scheme (LDAKM-EIoT).*

### 4.3. Dynamic Node Addition Phase

There is the chance that some of the IoT devices fail to work properly or might stop their working completely. Furthermore, there is the possibility that some of the IoT devices can be “physically stolen (capture)” by an adversary A. In order to draw out the useful data from the stolen IoT devices (i.e., session key, temporary and pseudo identities, and other credentials stored in their memory), henceforth, it becomes very important to add new IoT devices in the required area. LDAKM-EIoT provides a facility for the addition of new IoT devices and also cloud servers in the network at any time after initial installment.

#### 4.3.1. Dynamic IoT Device Addition

The upcoming procedure is needed to add a new IoT device, say $D_{ni}$, in the required part of the IoT environment.

**DD1.** The TA picks a new unique identity $ID_{D_{ni}}$ for the new IoT device $D_{ni}$ and computes its corresponding pseudo identity as $RID_{D_{ni}}$=h(n$||ID_{D_{ni}})$, temporal credentials $TC_{D_{ni}}=$$h(RID_{D_{ni}}$$||ID_{EN}$$||ID_{TA}$||n$||RTS_{D_{ni}})$, $AD_{ni}$$=h(K_{EN-D_{ni}}||$$ID_{D_{ni}}\left|\right|$$ID_{EN}\left|\right|$$RTS_{D_{ni}})$ where $RTS_{D_{ni}}$ is registration timestamp generated for $D_{ni}$ and $K_{EN-D_{ni}}$ is the shared secret key between IoT device $D_{ni}$ and the edge node EN generated by the TA. TA also generates a unique temporary identity $TID_{D_{ni}}$ for $D_{ni}$ that is different from the previously deployed IoT devices.

**DD2.** The TA stores the credentials {$RID_{D_{ni}},$$TID_{D_{ni}},$$TC_{D_{ni}},$$AD_{ni},$h(·)} in the memory of $D_{ni}$ before its placement in the deployment area. Furthermore, the TA securely sends the information $\{RID_{D_{ni}},$$TID_{D_{ni}},$$TC_{D_{ni}},$$AD_{ni}\}$ related to $D_{ni}$ to EN so that the EN can update these information in its database corresponding to the newly added IoT device $D_{ni}$. Note that for secure communication between TA and EN, a pre-shared symmetric key among them is essential, and it is generated by the TA prior to the placement of the EN.

#### 4.3.2. Dynamic Cloud Server Addition

The upcoming procedure is required to add a new cloud server, say $CS_{nj}$, in the required part of the IoT environment.

**DC1.** The TA chooses a distinct unique identity $ID_{CS_{nj}}$ for $CS_{nj}$ and computes its pseudo identity as $RID_{CS_{nj}}$=h(n$||ID_{CS_{nj}})$ and $ACS_{nj}$$=h(K_{EN-CS_{nj}}$$||ID_{CS_{nj}}$$||ID_{EN}$$||RTS_{CS_{nj}})$ where $RTS_{CS_{nj}}$ is the registration timestamp for $CS_{nj}$. Moreover, $K_{EN-CS_{nj}}$ is the shared secret key of the edge node EN and cloud server $CS_{nj}$ generated by the TA.

**DC2.** The TA then stores the credentials {$RID_{CS_{nj}},$$ACS_{nj},$$ID_{CS_{nj}},$h(·)} into the database of $CS_{nj}$ prior to its deployment in the IoT environment.

## 5. Security Analysis of LDAKM-EIoT

In this section, we provide the security analysis of LDAKM-EIoT in Propositions 1–7, which prove its robustness against the following possible attacks.

**Proposition** **1.** *LDAKM-EIoT is resilient against replay attack.*

**Proof.**  In LDAKM-EIoT, we utilized various current timestamp values for the communicating entities ($D_{i}$, EN, and $CS_{j}$). In the each exchanged message of LDAKM-EIoT, we used the maximum transmission delay ΔT factor, which is a small value. Consequently, an adversary A can not gain any advantage in replaying the old transmitted messages, which are used in the authentication and key management procedure among $D_{i}$, EN, and $CS_{j}$ within ΔT. Thus, LDAKM-EIoT is secure against replay attack. □

**Proposition** **2.** *LDAKM-EIoT is resilient against man-in-the-middle attack (MITM).*

**Proof.**  Suppose an adversary A seizes an authentication request message $\left\{TID_{D_{i}},M_{1},M_{2},T_{1}\right\}$ exchanged between $D_{i}$ and EN and further tries to update this message in such a way that it looks similar to a genuine authentication message, as $\{TID_{D_{i}}^{a},M_{1}^{a},$$M_{2}^{a},T_{1}^{a}\}$ by using the parameters such as $M_{1}^{a}\;$$=AD_{i}⊕r_{d_{i}}^{a}$ and $M_{2}^{a}\;$$=h(AD_{i}||T_{1}^{a}||$$TID_{D_{i}}^{a}\;||RID_{D_{i}}\left|\right|$$TC_{D_{i}}\left|\right|r_{d_{i}})$. The values of the used variables are $RID_{D_{i}}$$=h(n||ID_{D_{i}})$, $TC_{D_{i}}=h(RID_{D_{i}}||ID_{EN}||ID_{TA}||n||RTS_{D_{i}})$, $AD_{i}$$=h(K_{EN-D_{i}}||$$ID_{D_{i}}||ID_{EN}\left|\right|$$RTS_{D_{i}})$ where $RTS_{D_{i}}$ is the registration timestamp for $D_{i}$ and $K_{EN-D_{i}}$ is the shared secret key of IoT device and edge node generated by the TA. To launch this attack, A can initiate the generation of random nonce $r_{d_{i}}^{a}$ and current timestamp $T_{1}^{a}$. However, without obtaining the information about “long term secret” $RID_{D_{i}}$, $ID_{EN}$, $ID_{TA}$, *n*, and $K_{EN-D_{i}}$, A cannot re-create another valid authentication request message $\{TID_{D_{i}}^{a},M_{1}^{a},$$M_{2}^{a},T_{1}^{a}\}$. By using a similar approach, we can also explain that other messages cannot be recomputed by A, which are used in the “authentication and key management” phase without the “long term secrets” used by $D_{i}$, EN, and $CS_{j}$. This proves the resilience of LDAKM-EIoT against MITM. □

**Proposition** **3.** *LDAKM-EIoT provides protection against different impersonation attacks.*

**Proof.**  Suppose an adversary A attempts to create a valid authentication request message on behalf of a communicating party (for example, $D_{i}$) after obtaining $D_{i}$’s authentication request $Msg_{1}=\{TID_{D_{i}},M_{1},$$M_{2},T_{1}\}$ sent towards the EN by $D_{i}$, where $M_{1}$$=AD_{i}⊕r_{d_{i}}$ and $M_{2}$$=h(AD_{i}||T_{1}||$$TID_{D_{i}}||RID_{D_{i}}\left|\right|$$TC_{D_{i}}\left|\right|r_{d_{i}})$. The values of the used variables are $RID_{D_{i}}$$=h(n||ID_{D_{i}})$, $TC_{D_{i}}=h(RID_{D_{i}}||ID_{EN}||ID_{TA}||n||RTS_{D_{i}})$, and $AD_{i}$$=h(K_{EN-D_{i}}||$$ID_{D_{i}}||ID_{EN}\left|\right|$$RTS_{D_{i}})$ where $RTS_{D_{i}}$ is the registration timestamp for $D_{i}$ and $K_{EN-D_{i}}$ is the shared secret key of IoT device and edge node generated by the TA. Note that $Msg_{1}$ uses $M_{1}$ and $M_{2}$, which are computed using the “long term secrets”($RID_{D_{i}}$, $ID_{EN}$, $ID_{TA}$, *n*, $K_{EN-D_{i}}$) and also the “short term secrets” (random nonce $r_{d_{i}}$). Without knowing these secret values, A is not able to create a valid authentication request message on behalf of the genuine IoT device $D_{i}$. Hence, we can say that LDAKM-EIoT is resilient against IoT device impersonation attack. In the same way, it can also be proven that LDAKM-EIoT provides protection against edge node and cloud server impersonation attacks as the creation of other messages $Msg_{2}$, $Msg_{3}$, and $Msg_{4}$ employs both “long term” and “short term” secrets. Therefore, LDAKM-EIoT is secure against impersonation attacks. □

**Proposition** **4.** *LDAKM-EIoT protects Ephemeral secret leakage (ESL) attack.*

**Proof.**  In LDAKM-EIoT, the session key is computed by a genuine cloud server $CS_{j}$ and the accessed $D_{i}$ during the the authentication and key management phase as $SK_{CS_{j}-D_{i}}$$=h(TID_{D_{i}}||$$T_{3}\left|\right|$$h(r_{en_{1}}$$\left|\right|T_{1}$$||AD_{i}$$||ID_{D_{i}}$$\left|\right|r_{d_{i}}$$||TC_{D_{i}})$$||h(r_{CS_{j}}$$||ACS_{j}$$||ID_{CS_{j}}$$\left|\right|T_{3}))$. Here, $AD_{i}$$=h(K_{EN-D_{i}}||$$ID_{D_{i}}||ID_{EN}\left|\right|$$RTS_{D_{i}})$ where $K_{EN-D_{i}}$ is the shared secret key of the IoT device and edge node generated by the TA and $K_{EN-CS_{i}}$ is the shared secret key of the EN and cloud server generated by the TA. Further, the identities of the IoT device, $ID_{D_{i}}$, the identity of the edge node $ID_{EN}$, and the identity of the cloud server $ID_{CS_{j}}$ are also utilized. It is understandable that the “session key” is the combination of both the session temporary (ephemeral) information, also called “short term secrets” (different random nonces and timestamps), as well as the “long term secrets” (different secret keys and identities). Thus, the session key can only be disclosed if A compromises both the session temporary and long term secrets. Moreover, as different random nonces and timestamps are used in the computation of the session keys between $D_{i}$ and $CS_{j}$ in distinct sessions, even if a session key is disclosed for a specific session, it will not result in computing the session keys of other sessions because of the combination of short and long term secrets. Thus, LDAKM-EIoT provides protection against session temporary information attack, and it also preserves the “perfect forward secrecy” goal. Therefore, LDAKM-EIoT is resilient against “ESL attack”. Consequently, LDAKM-EIoT preserves the session key security under the “CK adversary model” [40]. □

**Proposition** **5.** *LDAKM-EIoT is resilient against privileged-insider attack.*

**Proof.**  The privileged-insider user of a cloud server, say A, knows the registration information {$RID_{CS_{j}},ACS_{j},ID_{CS_{j}},h\left(\cdot \right)$}. However, he/she cannot calculate the “session key” on behalf of the cloud server as the session key utilizes the credentials that are only known to the IoT devices or the edge node. The “session key” calculated by the legitimate cloud server is $SK_{CS_{j}-D_{i}}$$=h(TID_{D_{i}}||$$T_{3}\left|\right|$$h(r_{en_{1}}$$\left|\right|T_{1}$$||AD_{i}$$||ID_{D_{i}}$$\left|\right|r_{d_{i}}$$||TC_{D_{i}})$$||h(r_{CS_{j}}$$||ACS_{j}$$||ID_{CS_{j}}$$\left|\right|T_{3}))$. Moreover, $r_{en_{1}}$ is the random nonce of the EN. The identities of the IoT device, $ID_{D_{i}}$, the identity of the edge node $ID_{EN}$, and identity of the cloud server $ID_{CS_{j}}$ are also utilized in $SK_{CS_{j}-D_{i}}$. The privileged-insider user of the cloud server does not have any information about $ID_{D_{i}}$, $ID_{EN}$, and $K_{EN-D_{i}}$. Therefore, A cannot calculate the “session key” on behalf of the cloud server, as well as he/she cannot impersonate the cloud server, as explained in Proposition 3. Hence, LDAKM-EIoT is resilient against “privileged-insider attack”. □

**Proposition** **6.** *LDAKM-EIoT preserves the anonymity and untraceability properties.*

**Proof.**  Let us assume an adversary A intercepts the messages $Msg_{1}$$=\{TID_{D_{i}},$$M_{1},$$M_{2},$$T_{1}\}$, $Msg_{2}$$=\{TID_{D_{i}}^{\prime },$$M_{3},$$M_{4},$$T_{2}\}$, $Msg_{3}$$=\{M_{5},$$M_{6},$$M_{7},$$T_{3}\}$, and $Msg_{4}$$=\{M_{8},$α,β,$M_{9},$$M_{10},$$T_{3},$$T_{4}\}$ during the “authentication and key management phase” between $D_{i}$ and $CS_{j}$ via the EN. We used random nonces and current timestamps in the construction of messages, which helped to generate dynamic and unique messages for different sessions. Moreover, the identities $ID_{D_{i}}$, $ID_{EN}$, and $ID_{CS_{j}}$ are not transmitted in the plaintext format during transmission. This helps to attain both the anonymity and untraceability objectives of LDAKM-EIoT. □

**Proposition** **7.** *LDAKM-EIoT provides protection against IoT device physical capture attack.*

**Proof.**  Each IoT device stores the credentials {$RID_{D_{i}},$$TID_{D_{i}},$$TC_{D_{i}},$$AD_{i},$h(·)}, which are used for the “authentication and key management” purposes with other network communicating entities. The protection against IoT device physical capture attack is a very essential security requirement [50,51]. Suppose $n_{c}$ IoT devices are physically captured by an adversary A. We assess the IoT device physical capture attack as the fraction of total secure communications that are compromised from the capturing of $n_{c}$ IoT devices not including the communication in which the “compromised IoT devices” are straightforwardly extended. For example, one can estimate the probability of A’s ability to decrypt the secure communication between a cloud server $CS_{j}$ and a non-compromised IoT device $D_{i}$ when $n_{c}$ IoT devices are already physically stolen and compromised. Suppose this probability is represented by $P_{e}\left(n_{c}\right)$. If $P_{e}\left(n_{c}\right)=0$, an “authentication and key management” protocol is termed as “unconditionally secure against IoT device physical capture attack”. From a captured IoT device $D_{i}$, A will have the extracted credentials {$RID_{D_{i}},TID_{D_{i}},$$TC_{D_{i}},AD_{i},h\left(\cdot \right)$} along with the secret pairwise session key $SK_{CS_{j}-D_{i}}$ shared between $D_{i}$ and $CS_{j}$ from its memory. However, it is very important to notice that all $RID_{D_{i}}$, $TID_{D_{i}}$, $TC_{D_{i}}$, $AD_{i}$, $RTS_{D_{i}}$, and $K_{EN-D_{i}}$ are distinct for all installed IoT devices. Thus, the stolen $D_{i}$ by A can only help to find the secret keys between that $D_{i}$ and $CS_{j}$. Furthermore, this results in all other secret keys between that $CS_{j}$ and other non-compromised IoT devices $D_{i}$ to be not still revealed. Therefore, compromising an IoT device will not cause the compromise of the entire communication, and we get the secure communications among the cloud server and other non-compromised IoT devices. This means that LDAKM-EIoT is unconditionally secure against IoT device physical capture attack. □

## 6. Formal Security Verification Using AVISPA

This section shows the resilience of the proposed scheme (LDAKM-EIoT) against replay and man-in-the-middle attacks based on the formal security verification. We applied the broadly-accepted “Automated Validation of Internet Security Protocols and Applications (AVISPA)” tool [52] to achieve this goal. There are four backends, namely “on-the-fly mode-checker (OFMC)”, “constraint-logic based attack searcher (CL-AtSe)”, “SAT (Boolean satisfiability problem) based model checker (SATMC)”, and “tree automata based on automatic approximations for the analysis of security protocols (TA4SP)”, which are integrated with the AVISPA tool. These backends help in automatic execution analysis of the security protocols. The “High-Level Protocol Specification Language (HLPSL)” helps in converting the high level implementation of the the security protocols into the “Intermediate Format (IF)” using the HLPSl2IF translator. HLPSL defines the roles for all the involved participating entities in a protocol, which are termed as the basic roles. The compulsory roles, known as “session” and “goal and environment”, consist of the composition of the sessions along with globally defined constants, respectively. The IF is provided as input to one of the four backends to produce the “Output Format (OF)”, which explicitly explains under what conditions the protocol becomes “safe”, “unsafe”, or “inconclusive”. The precise discussion on AVISPA and its HLPSL is available in [52]. The AVISPA tool is one of the widely used formal security verification tools. Researchers working in the authentication domain frequently use this software tool to validate the security of authentication protocols [1,2,3,46,48,53].

**Figure 4:**
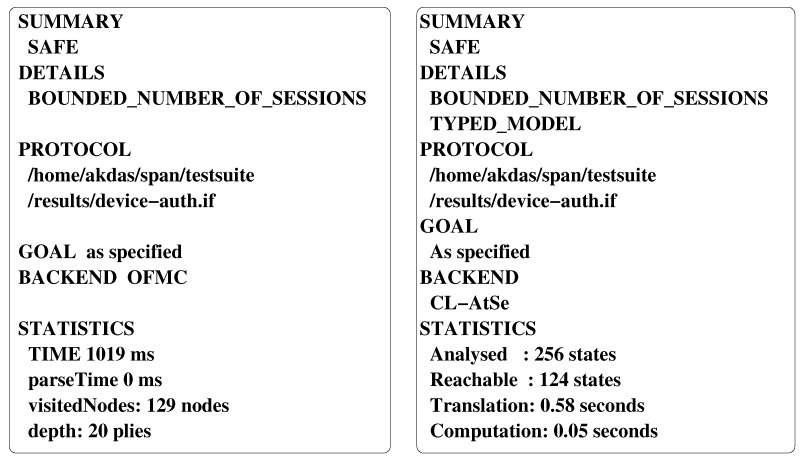
Analysis of simulation results under the CL-AtSe and OFMC backends.

In our implementation of the proposed scheme (LDAKM-EIoT) under HLPSL, we defined four basic roles: (a) the role for the TA, (b) the role for an IoT device $D_{i}$, (c) the role for the edge node EN, and (d) the role for a cloud server $CS_{j}$. Apart from these basic roles, we had two compulsory roles (“session” and “goal and environment”). We applied the “SPAN (Security Protocol ANimator for AVISPA)” [54] for simulating LDAKM-EIoT. The backends that we covered were OFMC and CL-AtSe, because they support the bitwise XOR operation. Therefore, we excluded the simulation results under other backends (i.e., SATMC and TA4SP) from the simulation part, because they did not currently support XOR operation, and the results would be under these backends “inconclusive”. Figure 4 shows the simulation results under the OFMC and CL-AtSe backends. The summary of these backends helped us to predict the security of a designed scheme against replay and man-in-the-middle attacks. Under the OFMC backend, the simulation took 1019 milliseconds, while traversing 129 visited nodes at a depth of 20 plies. On the other hand, under the CL-Atse backend, 256 states were analyzed, and out of those states, 124 states were reachable with 0.58 s of translation time and 0.05 s of computation. The provided simulation results assured that LDAKM-EIoT was safe against the replay and man-in-the-middle attacks.

## 7. Comparative Analysis with Existing Schemes

In this section, the comparative study of LDAKM-EIoT with other related existing schemes, such as the schemes designed by Challa et al. [3], Farash et al. [21], Sharma and Kalra [39], Zhou et al. [41], and Turkanovic et al. [18], is done. It is worth noting that the schemes of Challa et al. [3], Farash et al. [21], Sharma and Kalra [39], Zhou et al. [41], and Turkanovic et al. [18] were designed for the IoT environment.

### 7.1. Comparative Study of Communication Costs

We took the identity, random nonce, hash digest (if the secure hash algorithm (SHA-1) [55] was utilized), and timestamp as 160, 128, 160, and 32 bits, respectively. It well known that the 1024 bit “RSA public key cryptosystem” maintains the same security level as that for the 160 bit “elliptic curve cryptography (ECC) cryptosystem” [56]. Thus, an elliptic curve point of the form $P=(P_{x},P_{y})$ took (160+160)=320 bits. The communication costs of LDAKM-EIoT and other schemes are provided in Table 2. In LDAKM-EIoT, the messages $Msg_{1}$$=\{TID_{D_{i}},$$M_{1},$$M_{2},$$T_{1}\}$, $Msg_{2}$$=\{TID_{D_{i}}^{\prime },$$M_{3},$$M_{4},$$T_{2}\}$, $Msg_{3}$$=\{M_{5},$$M_{6},$$M_{7},$$T_{3}\}$, and $Msg_{4}$$=\{M_{8},$α,β,$M_{9},$$M_{10},$$T_{3},$$T_{4}\}$ incurred the costs of (160+160+160+32)=512 bits, (160+160+160+32)=512 bits, (160+160+160+32)=512 bits, and (160+160+160+160+160+32+32)=864 bits, respectively, and hence, all the messages needed a total of (512+512+512+864)=2400 bits. On the other side, the communication costs for the schemes of Challa et al. [3], Farash et al. [21], Sharma and Kalra [39], Zhou et al. [41], and Turkanovic et al. [18] were 2528, 2752, 2912, 3840, and 2720 bits, respectively. It is depicted in Table 2 that LDAKM-EIoT performed better in terms of communication cost as compared to other considered existing schemes of Challa et al. [3], Farash et al. [21], Sharma and Kalra [39], Zhou et al. [41], and Turkanovic et al. [18].

### 7.2. Comparative Study of Computation Costs

The notations $T_{ecm}$, $T_{fe}$, and $T_{h}$ are used to represent the time required for an “ECC point multiplication”, a “fuzzy extractor function” for biometric verification in the case of the scheme designed by Challa et al. [3], and a “one way hash function”, respectively. Based on the experimental results available in [57], we took $T_{h}$≈0.00032 s, $T_{ecm}$≈0.0171 s, and $T_{fe}$$\approx T_{ecm}$, that is $T_{fe}$≈0.0171 s. The computation costs’ comparison among LDAKM-EIoT and other schemes is depicted in Table 3. In LDAKM-EIoT, during the authentication and key agreement procedure, $D_{i}$, EN, and $CS_{j}$ incurred $9T_{h}$≈2.88 ms, $15T_{h}$≈4.8 ms, and $8T_{h}$≈2.56 ms, respectively. The comparative results demonstrated that LDAKM-EIoT performed better than Challa et al.’s scheme [3] and Zhou et al.’s scheme [41]. Moreover, LDAKM-EIoT needed the same computation cost as compared to that for Farash et al.’s scheme [21]. However, LDAKM-EIoT required more computation cost as compared to that for Sharma and Kalra’s scheme [39] and Turkanovic et al.’s scheme [18]. However, this was accepted as LDAKM-EIoT provided extra security and functionality features as compared to those for the other compared schemes of Challa et al. [3], Farash et al. [21], Sharma and Kalra [39], Zhou et al. [41], and Turkanovic et al. [18] (see Table 4).

### 7.3. Comparative Study of Security and Functionality Attributes

The comparison of the security and functionality attributes for the proposed LDAKM-EIoT and other related schemes is shown in Table 4. Various features ($FSF_{1}$-$FSF_{4}$, $FSF_{6}$, $FSF_{17}$, and $FSF_{18}$) were not supported/available in the scheme of Farash et al. [21], whereas Challa et al.’s scheme [3] did not support the features $FSF_{11}$ and $FSF_{14}$, which were shown by Jia et al. [37]. Moreover, other schemes, such as the schemes of Sharma and Kalra [39], Zhou et al. [41], and Turkanovic et al. [18] did not provide the required functionality and security features. On the other hand, LDAKM-EIoT provided better security and functionality features as compared to other related schemes.

## 8. NS2 Simulation Study

In this section, we conduct the simulation study on LDAKM-EIoT and other existing related schemes of Challa et al. [3], Farash et al. [21], Sharma and Kalra [39], Zhou et al. [41], and Turkanovic et al. [18] using the NS2 simulator. The impact of LDAKM-EIoT and other related schemes is studied and compared on important network performance parameters (for example, throughput (available in bits per second (bps)) and end-to-end delay (available in seconds)).

### 8.1. Simulation Parameters

The specifics of the network parameters used in the NS2 simulation are given in Table 5. The simulation time was 30 min (1800 s). $S_{j}$ and $CS_{j}$ respectively represent the *j*th sensor node and cloud server, whereas $U_{i}/D_{i}$ represents the *i*th user/IoT smart device respectively in LDAKM-EIoT and other schemes [3,21]. Moreover, we took one gateway node (GW)/edge node (EN) in all the compared schemes. The communication range of smart device or sensor was taken as 100 m. The messages exchanged among different communicating entities along with their communication costs (in bits) for different schemes are depicted in Table 6, which were used in the simulation for exchanging the packets (messages) among the entities. We applied the ad hoc on-demand distance vector (AODV) [58] as the routing protocol. All other parameters used in the simulation were taken as standard ones.

### 8.2. Comparative Analysis of the Simulation Results

The comparative analysis of the network performance parameters for LDAKM-EIoT, Challa et al. [3], and Farash et al. [21] is discussed in the following.

#### 8.2.1. Comparative Analysis of the Network Throughput

**Figure 5:**
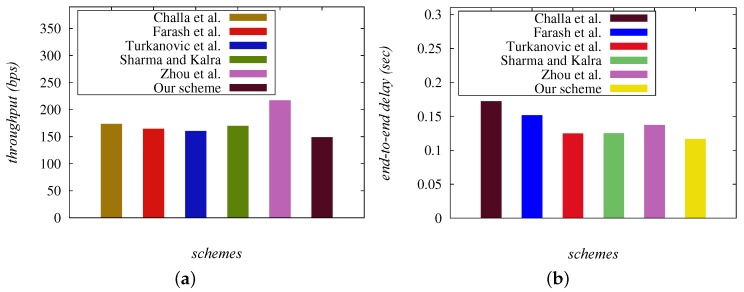
Comparison of network parameters: (**a**) network throughput; (**b**) end-to-end delay.

The network throughput can be calculated as “the number of bits transmitted per unit time, and it can be mathematically expressed as $(\nu_{r}\times \left|\rho |\right)/T_{d}$, where $T_{d}$ is the total time (in seconds), |ρ| the size of a packet, and $\nu_{r}$ the total number of received packets”. The simulation time was 1800 s, which was the actual total considered time. In Figure 5a, the throughput values for the schemes of Challa et al. [3], Farash et al. [21], Turkanovic et al. [18], Sharma and Kalra [39], Zhou et al. [41], and the proposed LDAKM-EIoT are 173.49 bps, 164.34 bps, 160.41 bps, 169.85 bps, 217.16 bps, and 148.95 bps, respectively. It was observed that the throughput of LDAKM-EIoT was less than the other schemes [3,18,21,39,41]. This was primarily due to the reason that LDAKM-EIoT applied the small sized messages, which caused less communication overhead for the authentication procedure as compared to the other schemes (see Table 6).

#### 8.2.2. Comparative Analysis of the End-to-End Delay

The end-to-end delay (EED) is measured as “the average time taken by the data packets to arrive at a receiving node from a sender node, and it is mathematically expressed in the form $\sum_{i=1}^{\nu_{p}}\left(T_{rec_{i}}-T_{send_{i}}\right)/\nu_{p}$, where $T_{rec_{i}}$ and $T_{send_{i}}$ are the receiving and sending time of a packet *i*, respectively, and $\nu_{p}$ the total number of packets”. From the simulation results provided in Figure 5b, it was observed that EED values for the schemes of Challa et al. [3], Farash et al. [21], Turkanovic et al. [18], Sharma and Kalra [39], Zhou et al. [41], and LDAKM-EIoT were 0.17230 s, 0.15151 s, 0.12478 s, 0.12496 s, 0.13707 s, and 0.11640 s, respectively. The EED value of LDAKM-EIoT was less than the schemes provided in [3,18,21,39,41]. This was again due to the reason that LDAKM-EIoT applied small sized messages for the authentication procedure, which caused less end-to-end delay as compared to other schemes.

## 9. Conclusions

The edge based IoT environment suffers from serious security and privacy issues. It was observed that the majority of the existing schemes for authentication and key management have limitations as they were vulnerable to various attacks. Some of them were not even efficient from the computation and communication cost point of view. Consequently, a new authentication and key management scheme with lightweight cryptographic operations was designed for the edge based IoT environment (LDAKM-EIoT). The rigorous security analysis of LDAKM-EIoT using formal security verification using AVISPA tool and also other security analysis evidenced that LDAKM-EIoT could prevent different possible well known attacks. LDAKM-EIoT also supported new smart IoT device deployment in the network after the initial deployment. Moreover, LDAKM-EIoT preserved the anonymity and untraceability properties. LDAKM-EIoT was also compared with the closely related existing schemes in the IoT environment. The conducted comparative performance analysis and NS2 based simulation study on the network performance parameters evidenced that LDAKM-EIoT was much better in security and functionality features, communication, and computation as compared to other existing schemes.

In the future, we would like to implement the proposed LDAKM-EIoT in a testbed environment. Moreover, we would also like to include more functionality features in the proposed LDAKM-EIoT while maintaining less communication and computational overheads without degrading the security.

## Figures and Tables

**Table 1:** Notations used in the lightweight authenticated key management mechanism for the edge-based IoT environment (LDAKM-EIoT).

Symbol	Meaning
$D_{i}$, $ID_{D_{i}}$	*i*th IoT device and its identity, respectively
$CS_{j}$, $ID_{CS_{j}}$	*j*th cloud server and its identity, respectively
EN, $ID_{EN}$	Edge node and its identity, respectively
TA, $ID_{TA}$	Trusted authority and its identity, respectively
$RID_{D_{i}}$, $RID_{CS_{j}}$	Pseudo identities of $D_{i}$ and $CS_{j}$, respectively
$TID_{D_{i}}$	Temporary identity of $D_{i}$
$K_{EN-D_{i}}$	1024 bit shared secret key of the IoT device and edge node generated by TA
$K_{EN-CS_{i}}$	1024 bit shared secret key of the edge node and cloud server generated by TA
*n*	160 bit secret secret of TA, which is only known to TA.
$RTS_{D_{i}}$	Registration timestamp of $D_{i}$
$n_{d},n_{c}$	Number of IoT devices and cloud servers deployed initially, respectively
$r_{d_{i}}$, $r_{en_{1}}$, $r_{en_{2}}$, $r_{cs_{j}}$	128 bit random numbers of $D_{i}$, EN, and $CS_{j}$, respectively
$T_{1},T_{2},T_{3},T_{4}$	Current timestamps generated by different entities
ΔT	“Maximum transmission delay” associated with a message
h(·)	“Collision resistant cryptographic one way hash function”
$SK_{D_{i}-CS_{j}}$	Session key between $D_{i}$ and $CS_{j}$
||, ⊕	Concatenation and bitwise XOR operations, respectively

**Table 2:** Communication costs’ comparison. LDAKM-EIoT, the lightweight authentication and key management scheme for the edge based IoT environment.

Protocol	No. of Messages	No. of Bits
LDAKM-EIoT	4	2400
Challa et al. [3]	3	2528
Farash et al. [21]	4	2752
Sharma and Kalra [39]	4	2912
Zhou et al. [41]	4	3840
Turkanovic et al. [18]	4	2720

**Table 3:** Comparison of computation costs.

Scheme	User/Smart	Gateway Node/	Sensing Device	Total Cost
	IoT Device	Edge Node	/Cloud Server	
LDAKM-EIoT	$9T_{h}$	$15T_{h}$	$8T_{h}$	$32T_{h}$
	≈2.88 ms	≈4.8 ms	≈2.56 ms	≈10.24 ms
Challa et al. [3]	$1T_{fe}+5T_{ecm}+$ $5T_{h}$	$5T_{ecm}+4T_{h}$	$4T_{ecm}+3T_{h}$	$1T_{fe}+14T_{ecm}+$ $12T_{h}$
	≈104.20 ms	≈86.78ms	≈69.36 ms	≈260.34 ms
Farash et al. [21]	$11T_{h}$	$14T_{h}$	$7T_{h}$	$32T_{h}$
	≈3.52 ms	≈4.48 ms	≈2.24 ms	≈10.24 ms
Sharma and Kalra [39]	$11T_{h}$	$7T_{h}$	$5T_{h}$	$23T_{h}$
	≈3.52 ms	≈2.24 ms	≈1.6 ms	≈7.36 ms
Zhou et al. [41]	$10T_{h}$	$7T_{h}$	$19T_{h}$	$36T_{h}$
	≈3.2 ms	≈2.24 ms	≈6.08 ms	≈11.52 ms
Turkanovic et al. [18]	$7T_{h}$	$5T_{h}$	$7T_{h}$	$19T_{h}$
	≈2.24 ms	≈1.6 ms	≈2.24 ms	≈6.08 ms

**Table 4:** Comparison of functionality and security features.

Feature	Farash	Challa	Turkanovic	Sharma and	Zhou	LDAKM-EIoT
	et al. [21]	et al. [3]	et al. [18]	Kalra [39]	et al. [41]	
$FSF_{1}$	×	✓	✓	×	✓	✓
$FSF_{2}$	×	✓	×	×	×	✓
$FSF_{3}$	×	✓	×	×	✓	✓
$FSF_{4}$	×	✓	×	×	✓	NA
$FSF_{5}$	✓	✓	✓	✓	✓	✓
$FSF_{6}$	×	✓	×	✓	×	NA
$FSF_{7}$	✓	✓	✓	✓	×	✓
$FSF_{8}$	✓	✓	✓	✓	×	✓
$FSF_{9}$	✓	✓	✓	✓	×	✓
$FSF_{10}$	✓	✓	✓	✓	✓	✓
$FSF_{11}$	✓	×	×	✓	✓	✓
$FSF_{12}$	✓	✓	✓	✓	NA	✓
$FSF_{13}$	✓	✓	✓	✓	×	NA
$FSF_{14}$	✓	×	✓	✓	NA	✓
$FSF_{15}$	NA	✓	×	×	×	NA
$FSF_{16}$	✓	✓	×	✓	✓	✓
$FSF_{17}$	×	✓	×	×	×	NA
$FSF_{18}$	×	✓	✓	×	×	✓

Note: $FSF_{1}$: the property to make the user or smart IoT device anonymous; $FSF_{2}$: protection for privileged-insider attack; $FSF_{3}$: protection for off-line password guessing attack; $FSF_{4}$: protection for stolen smart card or mobile device attack; $FSF_{5}$: protection for denial-of-service attack; $FSF_{6}$: protection for user impersonation attack; $FSF_{7}$: protection for replay attack; $FSF_{8}$: protection for man-in-the middle attack; $FSF_{9}$: achieves mutual authentication; $FSF_{10}$: achieves session key agreement; $FSF_{11}$: property to make messages untraceability; $FSF_{12}$: protection for sensor node/sensor/smart IoT device physical capture attack; $FSF_{13}$: presence of server independent password update phase; $FSF_{14}$: protection for sensor node/sensing device/smart IoT device impersonation attack; $FSF_{15}$: support biometric update phase; $FSF_{16}$: provide formal security verification using the automated software verification tool; $FSF_{17}$: presence of the smart card revocation phase; $FSF_{18}$: protection for session-key security under the CK adversary model. ×: insecure against a “specific attack” or a “particular feature” is not there; ✓: secure against a “specific attack” or a “particular feature” is present; NA: not applicable.

**Table 5:** Different parameters used in the simulation. AODV, ad hoc on-demand distance vector.

Parameter	Description
Platform	Ubuntu 14.04 LTS
Tool used	NS2 2.35
Number of gateway nodes/edge nodes	1
(whenever applicable)	
Number of users or IoT device	9
(whenever applicable)	
Number of sensors or cloud server	10
(whenever applicable)	
Simulation time	1800 s
Communication range of sensors/IoT devices	100 m
Routing protocol	AODV [58]

**Table 6:** Different messages exchanged among entities used in the simulation.

Exchanged Messages between Network Entities	Challa	Farash	Turkanovic	Sharma	Zhou	LDAKM-EIoT
	et al. [3]	et al. [21]	et al. [18]	and Kalra [39]	et al. [41]	
$U_{i}/D_{i}\to GW/EN$	992 bits	512 bits	672 bits	672 bits	800 bits	512 bits
$GW/EN\to SD_{j}$/$S_{j}/CS_{j}$	1024 bits	1024 bits	1024 bits	1024 bits	1600 bits	512 bits
$SD_{j}$/$S_{j}/CS_{j}\to GW/EN$	−	672 bits	576 bits	672 bits	960 bits	512 bits
$GW/EN\to U_{i}/D_{i}$	−	544 bits	448 bits	544 bits	480 bits	864 bits
$SD_{j}$/$S_{j}/CS_{j}\to U_{i}/D_{i}$	512 bits	−	−	−	−	−
